# Prediction and validation of protein–protein interactors from genome-wide DNA-binding data using a knowledge-based machine-learning approach

**DOI:** 10.1098/rsob.160183

**Published:** 2016-09-28

**Authors:** Ashley J. Waardenberg, Bernou Homan, Stephanie Mohamed, Richard P. Harvey, Romaric Bouveret

**Affiliations:** 1 Victor Chang Cardiac Research Institute, Darlinghurst, New South Wales 2010, Australia; 2 Children's Medical Research Institute, University of Sydney, Westmead, New South Wales2145, Australia; 3 St Vincent's Clinical School, University of Sydney, Westmead, New South Wales2145, Australia; 4 School of Biotechnology and Biomolecular Science, University of New South Wales, Kensington, New South Wales2052, Australia

**Keywords:** machine learning, protein–protein interactions, transcription factors, gene regulatory networks

## Abstract

The ability to accurately predict the DNA targets and interacting cofactors of transcriptional regulators from genome-wide data can significantly advance our understanding of gene regulatory networks. NKX2-5 is a homeodomain transcription factor that sits high in the cardiac gene regulatory network and is essential for normal heart development. We previously identified genomic targets for NKX2-5 in mouse HL-1 atrial cardiomyocytes using DNA-adenine methyltransferase identification (DamID). Here, we apply machine learning algorithms and propose a knowledge-based feature selection method for predicting NKX2-5 protein : protein interactions based on motif grammar in genome-wide DNA-binding data. We assessed model performance using leave-one-out cross-validation and a completely independent DamID experiment performed with replicates. In addition to identifying previously described NKX2-5-interacting proteins, including GATA, HAND and TBX family members, a number of novel interactors were identified, with direct protein : protein interactions between NKX2-5 and retinoid X receptor (RXR), paired-related homeobox (PRRX) and Ikaros zinc fingers (IKZF) validated using the yeast two-hybrid assay. We also found that the interaction of RXRα with NKX2-5 mutations found in congenital heart disease (Q187H, R189G and R190H) was altered. These findings highlight an intuitive approach to accessing protein–protein interaction information of transcription factors in DNA-binding experiments.

## 1. Introduction

Complex gene regulatory networks (GRNs) guide development and tissue homeostasis in all organisms. While gene regulation is complex, transcription factors (TFs) provide a key focus for effector function in GRNs as their specific DNA recognition sequence motifs (transcription factor binding sites, TFBSs) are hard-wired into the genome sequence [1,2]. TFs do not act in isolation, and the progression of diverse cellular programmes in development depends upon binding site specificity, cooperativity of multiple TFs and the recruitment of a diversity of cofactors [3–7].

Recently, machine-learning algorithms have been applied to genome-wide datasets to make novel predictions related to cardiac GRN function. These studies have focused on predicting muscle-specific enhancers from validated training sets [8,9] or identifying known and novel TFs governing heart precursor and organ development based on sequence-level discriminators (motif grammar) [10,11]. While such studies have demonstrated the power of machine-learning approaches for validating known enhancers and predicting novel enhancers based on motif grammar, these methods have not yet been systematically focused on the discovery and validation of novel TF protein interactors—therefore relatively few such transcriptional cofactors have come to light. Furthermore, while large numbers of TFs have been proposed to act through indirect DNA binding [12,13], the nature and role of cofactors that indirectly guide TFs to regulatory elements has not been clarified or systematically validated.

NKX2-5 is an NK2-class homeodomain TF related to *Drosophila* tinman, and its expression during mammalian development is regionally restricted to the cardiac fields and forming heart tube, as well as other organ-specific domains [14]. Consistent with a combinatorial model for TF specification of heart development, NKX2-5 acts cooperatively with other cardiac TFs whose expression is similarly regionally restricted, including GATA4, ISL1, TBX2/3/5/20, MEF2C and SRF. These factors are thought to form a cardiac collective or ‘kernel’ of TFs that show recursive wiring (many cross-regulatory interactions) [1] and which perform the executive functions of the cardiac GRN. NKX2-5 is essential for normal heart development and mouse embryos carrying homozygous NKX2-5 loss-of-function or severe point mutations show a rudimentary beating myogenic heart tube lacking specialized chambers, valves, septa and conduction tissues, with subsequent growth arrest and death at mid-gestation [15]. In humans, *NKX2-5* is also one of the most commonly mutated single genes in congenital heart disease (CHD), with heterozygous mutations causative for a spectrum of CHD phenotypes, most prominently atrial septal defects and progressive conduction block [15].

To expand our knowledge of the cardiac GRN, we recently identified NKX2-5 targets in cultured HL-1 atrial cardiomyocytes using DNA-adenine methlyltransferase identification (DamID), a sensitive enzymatic method for detecting genome-wide protein–DNA interactions [16]. DamID complements the chromatin immunoprecipitation (ChIP) method for detection of TF-DNA interactions while avoiding some of the artefacts associated with chromatin cross-linking and use of poor quality antibodies [16,17]. Approximately 1500 target peaks were detected and, consistent with a role for NKX2-5 in normal heart development, proximal target genes were enriched for those involved in cardiac development and sarcomere organization.

Further analysis of our DamID data [16] and ChIP data [11] identifying genome-wide cardiac TF target sets suggests that cardiac kernel TFs collaborate and interact widely with each other and with many broadly expressed signal-gated DNA-binding TFs. This includes factors embedded within canonical signalling pathways such as SMAD and TCF proteins (downstream of BMP and WNT signalling, respectively), known to regulate cardiogenesis, as well as other extracellular signal-gated TFs of the ETS, TEAD, NFAT, STAT, YY, SP, LMO and MEIS families [8,18–21]. A model in which regionally restricted kernel TFs cooperate with broadly expressed but signal-gated TFs to define an organ-specific context for developmental programmes is compelling because it allows for great regulatory flexibility, consistent with the GRN model [1].

In this study, we applied machine-learning algorithms to generate models for wild type (WT) NKX2-5 targets based on motif grammar, exploiting replicate NKX2-5 DamID experiments performed 2 years apart [16]. We developed a knowledge-based lasso method to generate sparse models with very high concordance between experiments. Using this approach, we defined 27 TFs as discriminators of NKX2-5 DamID targets that included NKX2-5 and related proteins, as well as known direct NKX2-5 protein interactors such as TBX5, GATA1 and HAND1. We also identified novel NKX2-5 target discriminators and validated retinoid X receptor (RXRα), paired-related homeobox (PRRX2), Ikaros zinc fingers (IKZF1) and a number of their paralogues (PRRX1a, PRRX1b, IKZF3 and IKZF5) as direct NKX2-5 interactors using the yeast two-hybrid assay. Furthermore, we found that interactions between RXRα and a subset of NKX2-5 mutations causative for congenital heart disease (Q187H, R189G and R190H) were altered, linking TF–TF interaction networks to heart disease. To our knowledge, these are the first experiments to mine genome-wide TF–DNA interaction data for systematic discovery and validation of TF protein–protein interactions (PPIs) for expanding TF interactomes.

## 2. Results

### 2.1. Classification of bound regions by motif composition

We previously identified 1536 and 1571 NKX2-5 target peaks, respectively, in two DamID experiments performed 2 years apart [16]. Three and four replicates, respectively, contributed to peak selection in these experiments, which we refer to as NKX2-5_1_ and NKX2-5_2_ [22]. The peak overlaps between NKX2-5_1_ and NKX2-5_2_ were highly significant (*p* < 0.001) and comparison of gene ontology (GO) terms using a log odds ratio statistic implemented in the CompGO R package demonstrated these experiments were identical at a GO level [22].

**Figure 1.**
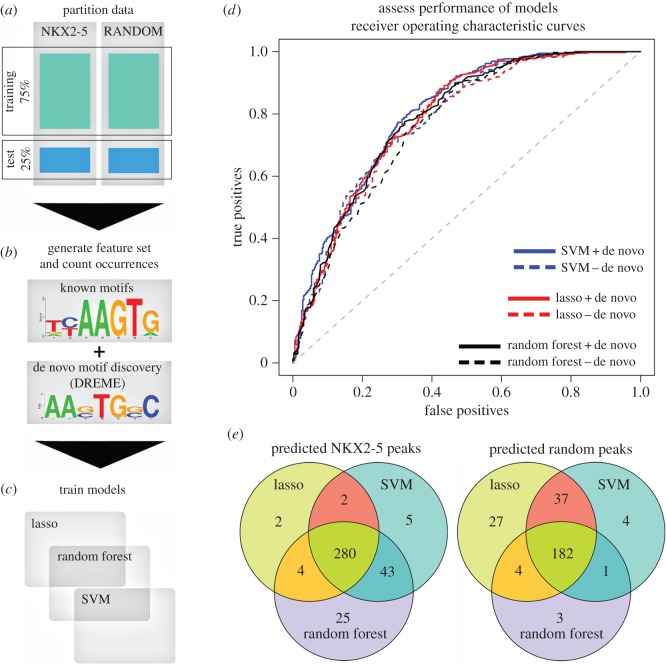
Predictive workflow for identification of novel PPIs. The process of building and testing models is illustrated as four steps from (*a*) to (*d*). (*a*) Partition data: NKX2-5 and random peaks were randomly partitioned into training (75%) and test (25%) sets. (*b*) Generate feature set and count occurrences: PWMs were determined de novo from training sets only and appended to an existing set of known PWMs. Motif occurrence was determined using CLOVER and normalized to peak length. (*c*) Train models: lasso, random forest and SVM models were trained and therefore generated from feature set data. (*d*) Evaluate models: receiver operator characteristic (ROC) curves for the lasso, random forest and SVM algorithms evaluated against the withheld test set. Blue lines, SVM; red lines, lasso; black lines, random forest; dashed lines correspond to removal of de novo motifs. (*e*) Overlap of test peaks correctly predicted as NKX2-5 positive or random using the lasso, SVM and random forest methods.

We sought to determine if NKX2-5 targets could be classified based on the motif grammar embedded within their peaks, relative to a random peak set generated from sequences represented on the Affymetrix promoter microarray chip used for DamID experiments [16]. For testing models, we used a leave-one-out cross-validation (LOOCV) approach to train models for NKX2-5_1_ (compared with the randomly generated peak set) on 75% of the data, withholding 25% for testing performance (figure 1*a*). We used DREME [23] to generate position weight matrices (PWMs) de novo from the training sets only, which identified 70 de novo PWMs in total (figure 1*b*; electronic supplementary material, file S1). We next combined these de novo PWMs with PWMs from Transfac [24] and Jaspar [25], adding the PWM for TBX5, a known NKX2-5 cofactor [26] (figure 1*b*; electronic supplementary material, file S2), bringing the total number of PWMs to 1202. CLOVER [27] was next used to count motif instances in NKX2-5 target peaks, followed by normalization to peak length. From de novo motifs discovered, the NKX2-5 motif (NKE) was highly enriched (ranked first for NKX2-5_1_ and shown in figure 1*b*, and third for NKX2-5_2_), consistent with previous findings [16].

We then generated classification models using three algorithms—least absolute shrinkage and selection operator (lasso) [28], support vector machine (SVM) [29] and random forest [30] (figure 1*c*)—and compared predictive performance using the area under curve (AUC) of receiver operating characteristic (ROC) graphs from the withheld test set (figure 1*d*). Performance of the lasso model on the test set resulted in an AUC of 0.789 (where 0.5 is random) (figure 1*d*). Removing de novo motifs from the feature matrix and refitting the model reduced the AUC slightly to 0.779—a marginal loss of 1% classification performance. AUCs of the SVM and random forest models using all motifs were 0.801 and 0.788, respectively, a marginal AUC improvement (0.012) or loss (−0.001) compared with the lasso model (figure 1*d*). Removing de novo motifs again did not affect performance (figure 1*d*). This indicated that known TFBSs were sufficient to predict NKX2-5 peaks. NKX2-5 test peaks predicted correctly by the SVM and random forest models overlapped highly with the lasso predicted peaks, identifying a common set of 280 positive peaks (approx. 78%) (figure 1*e*). Specificity and sensitivity analysis (equations (5.1) and (5.2); see Material and methods) revealed that the random forest was the most sensitive, predicting 88.7% as true positive peaks compared with the SVM (83.1%) and lasso (72.5%) (equation (5.1)). However, this was at the cost of specificity. The random forest model predicted the smallest proportion of true negative peaks (53.6%), followed by the SVM (63.2%) and lasso (70.6%) (equation (5.2)), suggesting a larger proportion of false positive predictions by the random forest and SVM models, and possibly overfitting by these models. Although these trade-offs were reflected by the overall similarity of their AUC values in the ROC curves (figure 1*d*), the lasso had the greatest positive predictive value (PPV, equation (5.3)), correctly predicting the largest proportion (73.5%) of true positive peaks among all positive predictions, followed by the SVM (71.7%) and random forest models (68.2%). Consistent with these results, the lasso model had the lowest false discovery rate (FDR) (equation (5.4); see Material and methods) at 0.265, followed by the SVM (0.283) and random forest (0.318). As our aim was to identify new PPIs with greatest confidence for further validation, we proceeded with the lasso algorithm, having the greatest PPV and the lowest FDR.

### 2.2. Assessing repeated NKX2-5 DamID binding experiments

**Figure 2.**
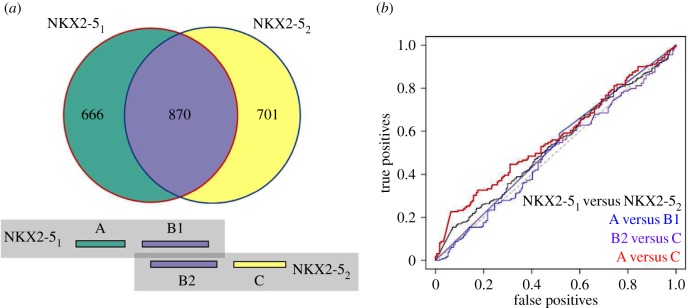
Classification accuracy of unique and common genomic regions from repeated experiments. (*a*) Peak overlaps of NKX2-5_1_ and NKX2-5_2_. Coloured boxes beneath match the venn diagram colours and represent the A, B1, B2 and C defined peaks, where A are unique peaks to NKX2-5_1_, B1 are the NKX2-5_1_ peaks that overlapped with NKX2-5_2_ peaks (vice versa for B2) and C are unique peaks to NKX2-5_2_. (*b*) ROC curves illustrating test set performance of models generated from direct comparison of NKX2-5 experiments as well as overlapping and non-overlapping genomic coordinates.

We then examined the similarity between distinct NKX2-5 experiments by applying lasso models generated from NKX2-5_1_ to test peaks obtained from NKX2-5_2_ and vice versa, and assessed sensitivity of predictions (equation (5.1)). The sensitivity of the NKX2-5_1_ lasso model for correctly predicting NKX2-5_2_ peaks was 0.741 if de novo motifs were included and 0.731 without de novo motifs. It is noteworthy here that the sensitivity of prediction was much greater than the approximately 55% of peak coordinates identified as overlapping between NKX2-5_1_ and NKX2-5_2_ (figure 2*a*) (see below).

To determine the importance of unique as well as common genomic targets in the overlap depicted in figure 2*a* for generating our models, data were split into A, B1, B2 and C sets, where A represented the peaks unique to NKX2-5_1_, B1 represents the specific peaks originating from NKX2-5_1_ which overlap with NKX2-5_2_ peaks, B2 the peaks originating from NKX2-5_2_ which overlap with NKX2-5_1_ peaks, and C the peaks unique to NKX2-5_2_. Positive predicted peaks (including de novo motifs) for each set of peaks followed: A (75.1%), B1 (80.2%), B2 (79.2%) and C (69.5%). An important insight here is that the non-overlapping A and C sets do not represent a random signature, consistent with our previously described GO term analysis of repeated NKX2-5 experiments, which showed that peaks unique to each experiment were enriched in similar GO categories [22]. Overlapping peaks (B sets) did, however, demonstrate improved sensitivity by 5–10%, and there was a small bias towards unique peaks (A versus C sets) from the experiment that the model was generated from. Results were consistent when excluding de novo motifs and when models were generated from NKX2-5_2_ versus random peaks and applied to A, B and C sets. However, the model for NKX2-5_2_ was much larger, with 142 features compared with 71 features for the NKX2-5_1_ model, and the number of positive peaks was slightly higher: A (71.2%), B1 (84.5%), B2 (83.6%) and C (77.6%).

It seemed unlikely that the increased number of features in NKX2-5_2_ could be explained by unique binding qualities of NKX2-5 between the two experiments or fundamental differences in the target cell states, given the similar number of peaks detected in NKX2-5_1_ and NKX2-5_2_, as well as the complete overlap in GO terms. The differences may rather relate to the inclusion or exclusion of borderline motifs in the models. To explore this further, we built models to compare each experiment directly (in contrast to comparing each to a random set) and all combinations of A, B and C (figure 2*a*) seeking features that might be unique to each experiment. Using the NKX2-5_1_ training set versus the NKX2-5_2_ training set, a classification model generated only four motifs (of which three were de novo) and the model performed poorly when applied to the withheld test data (AUC of 0.534) (figure 2*b*). Similarly, for A versus B, B versus C and A versus C, small models were generated (1, 1 and 6 motifs, respectively) with poor AUC performance (0.516, 0.569 and 0.508, respectively). The motifs present in the A versus C model were also present in the NKX2-5_1_ versus NKX2-5_2_ model (electronic supplementary material, figure S1). These results demonstrate that both NKX2-5 experiments, including their unique peaks, consistently captured peaks with similar TF binding site composition. Notably, the A and C peak sets showed similar features and are not artefacts (see Discussion).

### 2.3. Prediction of NKX2-5 protein : protein interactions

Our aim to experimentally detect and validate novel PPIs requires that we predict the smallest number of high-confidence targets for experimental follow-up. Because the lasso algorithm selects features by shrinking less relevant coefficients to zero through application of a *λ* penalty (via L-1 regularization; the shrinkage parameter), we investigated the model characteristics further. Removing de novo motifs (i.e. using only previously described motifs) and refitting a model for NKX2-5_1_ reduced the model size from 71 to 51 features (reduction of model size to approx. 72%) while only reducing classification performance by approximately 1% (AUC of 0.779). Considering this marginal loss of performance and the potential difficulty in associating de novo sequences to their cognate TFs, we continued to investigate the model based on previously described motifs. In addition, noting that the number of motifs included in the NKX2-5_2_ model was much larger than that for NKX2-5_1_ (112 versus 51), investigation of the *λ* curves revealed two differently shaped curves that resulted in a sparser model for NKX2-5_1_ (smaller number of features before reaching the 1 s.e. point of model selection) compared with NKX2-5_2_ (electronic supplementary material, figure S2).

**Figure 3.**
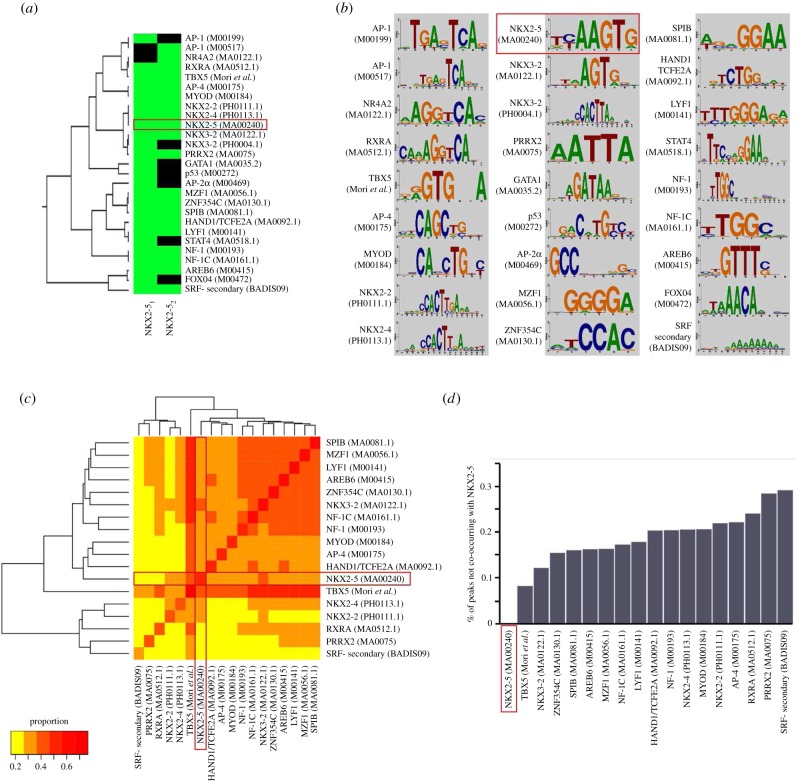
Knowledge-based model features. (*a*) Motifs remaining in NKX2-5_1_ and NKX2-5_2_ models after knowledge-based lasso modelling; green indicates present as a feature, black not present. Hierarchical clustering was performed in STAMP [32] using default parameters, except trimming was disabled. (*b*) Motifs of the knowledge-based models. (*c*) Co-occurrence matrix of motifs in common between NKX2-5_1_ and NKX2-5_2_. Scale 0–1 indicates proportion of total peaks in NKX2-5_1_ data that contained both motifs. (*d*) Proportion of peaks with a positive match for the motif of interest that did not co-occur with NKX2-5; NKX2-5 is therefore zero. Red boxes are to highlight the high-affinity NKX2-5 motif.

We therefore developed a ‘knowledge-based’ lasso method to reduce model size further, assessing the concordance of the models derived from replicate experiments. We hypothesized that the smallest predictive model for NKX2-5 would include NKX2-5 itself—the knowledge. We therefore continued to compress the lasso model until the point just before the known high-affinity NKX2-5 motif (‘NKX2-5 (M00240, Transfac)’; NKE) [31] was lost from our model (*λ* of approx. 0.071; electronic supplementary material, figure S2*a*). This resulted in a model with 25 features for NKX2-5_1_ (including a cluster of four motifs for other NK2-class homeodomain family members NKX2.2, 2.4 and 3.2, possibly representing alternative forms of the NKX2-5 TFBS), halving the model size and decreasing test AUC performance by only 0.020 (AUC 0.759). Similar results were achieved for the NKX2-5_2_ experiment versus random (*λ* of approx. 0.078; electronic supplementary material, figure S2*b*), where 18 of 20 features overlapped with the 25 features from the NKX2-5_1_ knowledge-based model (figure 3*a*,*b*). Notably, our knowledge-based model was no longer biased towards experimental origin, although overlap bias remained—that is, peaks common to NKX2-5_1_ and NKX2-5_2_ (represented as B1/B2 set; figure 2*a*) were predicted correctly more often: A (67.3%), B1 (75.1%), B2 (75.2%) and C (67.7%). This suggests that the knowledge-based approach was superior in eliminating model origin bias, consistent with results from fitting models directly against each other.

Many of the motifs included in this knowledge-based model were known NKX2-5 protein–protein interactors [16,33], speaking to the validity of the proposed approach. Motifs for known NKX2-5 PPIs common to both models included T-Box 5 (TBX5) [26], heart and neural crest derivatives expressed (HAND; MA0092.1), SP-1/ETS TFs (MA0081.1), and nuclear factor I (NF-I; MA0161.1 and M00193) [16]. NKX2-5_1_ but not NKX2-5_2_ features included known NKX2-5 interactors—the GATA binding protein (GATA1; MA0035.2) and tumour suppressor protein p53 (p53; M00272) [34,35]. Potentially novel NKX2-5 PPIs included myeloid zinc finger 1 (MZF1;MA0056.1), MYOD myogenic differentiation factor (MyoD;M00184), jun proto-oncogene (JUN/AP-1; M00199), TF AP-2/AP-4 (AP2/4; M00469, M00175), retinoid X receptor α (RXRα; MA0512.1), IKAROS family zinc finger 1 (IKZF1/LYF-1; M00141), zinc finger E-box binding homeobox 1 (ZEB1, also called AREB6; M00415) and paired-related homeobox 2 (PRRX2; MA0075.1).

Our knowledge-based models allow the possibility that WT NKX2-5 binding to DNA can be mediated by indirect as well as direct tethering to chromatin, as demonstrated for NKX2-5 mutant proteins [16], and thereby are potentially predictive of novel NKX2-5 PPIs that mediate indirect binding. We would expect, therefore, that a proportion of detected motifs would not co-occur with the NKX2-5 motif (NKE) in peaks. We assessed motifs common to both experiments and their frequency in NKX2-5_1_ peaks (figure 3*c*). Note that in figure 3*c* the proportions along the diagonal can be less than one, as this indicates prevalence of the TFBS among all peaks detected. With the exception of TBX5, which was present in a high proportion of NKX2-5 peaks, this analysis did not reveal a strong co-occurrence of NKX2-5 with the other TF binding sites detected. Subtracting NKX2-5 motif frequency from the frequency of other motifs detected revealed that, for each predicted motif, approximately 8–29% of peaks containing these motifs did not co-occur with the high-affinity NKX2-5 motif (figure 3*d*). These results support the hypothesis that NKX2-5 can bind to a subset of targets indirectly via PPIs.

The high co-occurrence of TBX5 motifs and the majority of other discriminators may have biological relevance [36], although may also reflect a relatively low information content of the TBX5 PWM.

### 2.4. Testing novel NKX2-5 protein–protein interactions

Identification of motifs in our lasso models that occur frequently in the absence of the high-affinity NKX2-5 TFBS suggests that TFs binding to these motifs might associate with NKX2-5 via PPIs to either recruit NKX2-5 specifically to these sites or interact on enhancers as part of higher order protein complexes. Searching PPI databases IntAct [37], HPRD [38], STRING [39] and BioGRID [40] for the term ‘NKX2-5’ revealed a small network of 31 known interactions (electronic supplementary material, figure S3 and table S1), from which we identified GATA4, SRF, TBX5 and HAND1 in our lasso models. Our model outputs suggest that there are many other possible NKX2-5 PPIs relevant to the cardiac GRN. Previous work focusing on the broadly expressed signal-gated ETS family TFs, ELK1 and ELK4, which are directly interacting cofactors of NKX2-5, showed that these were highly integrated in the cardiac GRN with many cross-regulatory interactions [16].

### 2.5. Testing novel PPIs

**Figure 4.**
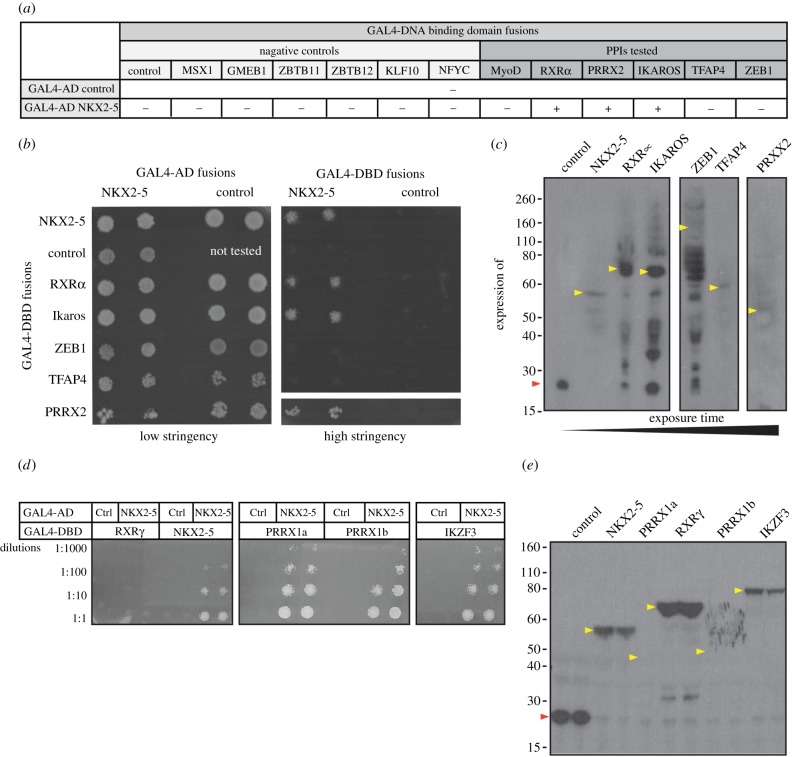
Validation of predicted PPIs by yeast-2-hybrid assay (Y2H). (*a*) Yeast transformed with the GAL4-activation domain (AD) alone, control, or fused to NKX2-5 and the GAL4-DNA-binding domain (DBD) alone (control) or fused to potential NKX2-5 protein interactors. Positive signs (+) show interaction as growth on selective medium (high stringency, -Leu/-Trp/-Ade/-His). (*b*) Representative picture of PPIs tested. Transformants were grown on non-selective (low stringency, -Leu/-Trp) before two clones were picked onto plates containing low or high stringency medium. (*c*) Detection of GAL4-DBD-Myc fusions by western blotting with anti-Myc antibodies. (*d*) Representative picture of PPIs tested with paralogues of novel NKX2-5 PPIs fused to the GAL4-DBD and grown on plates containing selective medium at four dilutions. (*e*) Detection of GAL4-DBD-Myc fusions by western blotting with anti-Myc antibodies. Coloured arrowheads indicate the expected molecular weight of control (red) or potential interactors (yellow).

To test whether unexpected motifs predicted in NKX2-5 targets correlate with novel NKX2-5 protein–protein interactors, we used the Y2H assay [16]. We fused NKX2-5 to the GAL4-activation domain (GAL4-AD) and its potential protein interactors to the GAL4 DNA-binding domain (GAL4-DBD). We initially tested six predicted and potentially novel PPIs from the knowledge-based model (RXRα, PRRX2, IKZF1, TFAP4, MyoD and ZEB1) and compared results to a set of TFs derived from randomly selected PWMs from the 1132 motifs used in this study (msh homeobox MSX1; glucocorticoid modulatory element binding protein 1 GMEB1; zinc finger and BTB domain containing 11 and 12 ZBTB11/12; Kruppel-like factor 10 KLF10; and nuclear TF Y-γ NFYC). We also included control vectors for expression of the GAL4-DBD and -AD alone. Using the Y2H assay under selective conditions, we confirmed that NKX2-5 fused to GAL4-AD bound specifically to GAL4-DBD fusions containing NKX2-5 itself, RXRα, PRRX2 or IKZF1/LYF, but not TFAP4/AP-4, ZEB1, MyoD or to any of the negative controls (figure 4*a*,*b*; electronic supplementary material, figure S4*a*). GAL4-DBD fusion expression was assessed by western blot using an antibody specific to the c-MYC tag present in the DBD fusions, which confirmed that RXRα, PRRX2, IKZF1 and TFAP4, as well as all negative controls tested, were expressed at the expected molecular weight (MW) in yeast (figure 4*c*; electronic supplementary material, figure S4*b*). ZEB1, however, showed little if any full-length protein and several degradation products, possibly due to its larger size (approx. 144 kDa). We therefore sub-cloned five overlapping sub-fragments of ZEB1/AREB6 spanning the whole protein (electronic supplementary material, figure S4*c*). Although expressed at the expected MWs, the N- and C-terminal ZEB1/AREB6 fragments, which contain the Zinc-finger clusters, failed to interact with NKX2-5 (electronic supplementary material, figure S4*c*,*d*). When fused to the GAL4-DBD, all the fragments encompassing the ZEB1 homeodomain resulted in background yeast growth, even when expressed with GAL4-AD alone. Therefore, PPIs between NKX2-5 and fragments containing the ZEB1 homeodomain could not be assessed properly in this system. Our results validated direct PPIs for three of the six TFs newly predicted from the knowledge-based model to bind to NKX2-5 targets, expanding the known NKX2-5 PPI network by 10% in this small validation screen. When considering previously known NKX2-5 PPIs also present in the model, we estimate that our predictive performance is in the range of approximately 60% (when including those untested as negative) to 80% (when not considering those untested).

### 2.6. Testing paralogues of novel NKX2-5 PPIs

Paralogous TFs are often reported to bind the same TFBS [41]. We therefore hypothesized that the motifs predicted by our model could represent binding of paralogous TFs for which no PWM was currently available, and so we extended our NKX2-5 PPI screen to paralogues of RXRα, PRRX2 and IKZF1. We found that NKX2-5 fused to GAL4-AD interacted with GAL4-DBD fusions containing transcript variants PRRX1a and PRRX1b, which are overall approximately 60% identical and paralogous to PRRX2 (figure 4*d*,*e*). Furthermore, IKZF3 (also known as Aiolos; figure 4*d*,*e*) and IKZF5 (also known as Pegasus; electronic supplementary material, figure S4*e*), which are overall 55% and 22% identical, respectively, to IKZF1, could also bind NKX2-5. These interactions are likely to occur through the first zinc-finger of the C-terminal dimerization domain (electronic supplementary material, figure S4*e*). However, while NKX2-5 interacted with RXRα, it did not interact with its paralogue RXRγ, which is overall 60% identical to RXRα.

### 2.7. Disease relevance of novel NKX2-5 PPIs

**Figure 5.**
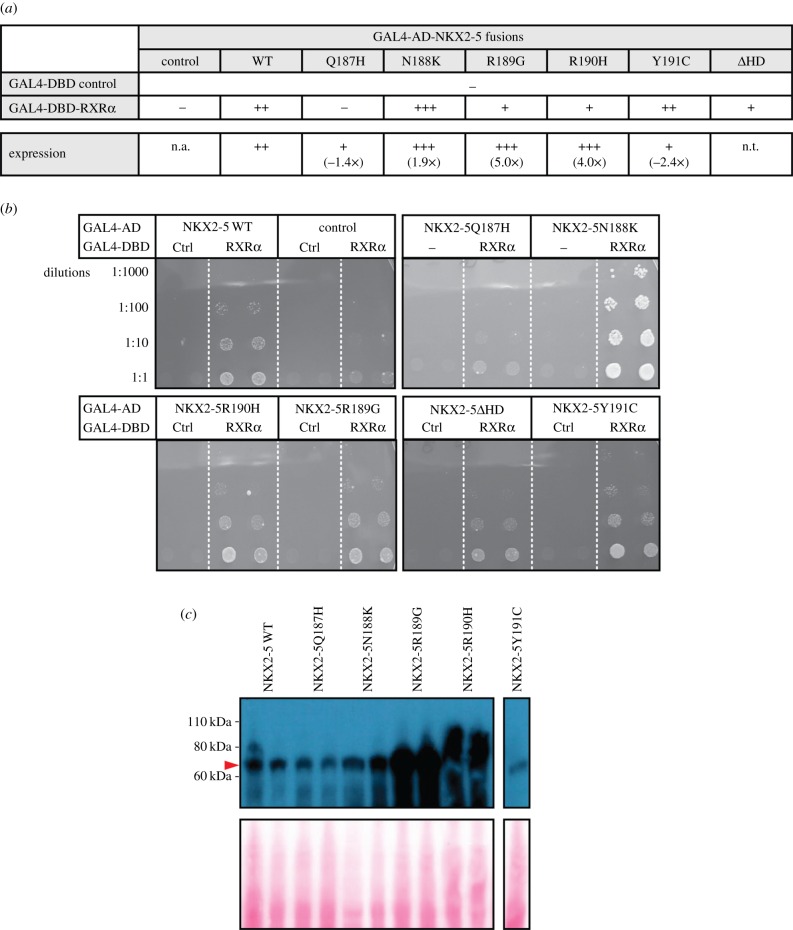
Testing PPIs between RXRα and NKX2-5 wild type (WT) or mutant yeast two-hybrid assay (Y2H). (*a*) Yeast transformed with the GAL4-activation domain (AD) alone (control) or fused to NKX2-5 proteins and the GAL4-DNA-binding domain (DBD) alone (control) or fused to RXRα. Positive signs (+) show interaction as growth on selective medium (high stringency, -Leu/-Trp/-Ade/-His). (*b*) Representative pictures of PPIs tested. Transformants were grown on non-selective (low stringency, -Leu/-Trp) before two clones were picked onto plates containing high-stringency medium. (*c*) Detection of two clones expressing GAL4-DBD-Myc fusions by western blotting with anti-Myc antibodies. The red arrowhead indicates the expected molecular weight of NKX2-5 proteins. The expression was quantified using ImageJ and the fold change relative to NKX2-5 WT is indicated in (*a*) (n.t., not tested).

Having identified novel PPIs, we next determined whether known CHD-causing mutations in NKX2-5 demonstrated impaired or altered binding to these PPIs. Mice lacking RXRα display a large spectrum of severe cardiac defects, including abnormal septation, ventricular phenotypes resulting from lack of expansion of the compact zone of myocardium and dysregulated trabecular morphogenesis [42], and these overlap with defects seen in NKX2-5 heterozygous and hypomorphic models [43–45]. We therefore tested a panel of five NKX2-5 point mutations in the homeodomain associated with heart disease: Q187H, N188 K, R189G, R190H and Y191C [46–48]. Three of the five mutants (Q187H, R189G and R190H) demonstrated a decreased interaction with RXRα when compared with NKX2-5 WT (figure 5*a*,*b*). For Q187H, this could be attributed to the lower expression observed in yeast as determined by western blotting (figure 5*a*,*c*). However, for R189G and R190H, expression in yeast was higher compared with that of WT, indicative of a true impairment of the PPI. Surprisingly, N188K interacted more strongly with RXRα than NKX2-5 WT, possibly because it showed increased expression or stability (figure 5*a*,*c*). These results suggest that the novel NKX2-5 PPI with RXRα identified here is critical for normal heart development and is disrupted in CHD caused by NKX2-5 homeodomain mutations.

## 3. Discussion

The ability to accurately predict DNA targets and interacting cofactors of transcriptional regulators from genome-wide data can significantly advance our understanding of GRNs and processes underlying disease. Here, we sought to determine if DNA regions detected as bound by NKX2-5, a TF essential for heart development, could be used to predict novel NKX2-5 protein interactors. We then systematically tested novel candidate PPIs and their paralogues for binding to NKX2-5, and, in the case of RXRα, for binding to NKX2-5 mutants using the yeast two-hybrid assay.

Exploiting NKX2-5 DNA-binding experiments that were repeated 2 years apart [16], we first investigated reproducibility by applying machine-learning algorithms to explore TFBS patterns in each experiment. This led to the development of a knowledge-based method for variable selection (i.e. model shrinkage), based on our assumption that a minimal model should contain NKX2-5 itself. Our knowledge-based method significantly improved model concordance between the repeat experiments. Models generated for each experiment correctly predicted bound regions of the other experiment with up to 80% accuracy compared with randomly selected peaks, far greater than the approximately 55% overlap of genomic coordinates (figure 2*a*). Concordance of our knowledge-based models from each experiment (figure 3*a*) reflected an underlying consistency of motif grammar, consistent with our previous finding of identical enriched GO terms between NKX2-5_1_ and NKX2-5_2_ target gene sets [22]. This suggests that each repeated experiment captures a unique subset of NKX2-5 binding sites that nonetheless have similar underlying motif composition. It is plausible that cells could have been exposed to slightly different environments (e.g. culture serum batch), representing a cell non-autonomous influence, albeit one that does not alter the binding logic of NKX2-5 or instigate global changes in GO terms of targeted genes. It has been proposed that alternative states exist within GRNs that contribute to robustness [49]. Typically, this concept has been used to explain variability inherent in signal transduction circuits [50], gene or protein expression variability [51] and, conversely, constraints or non-robustness that lead to disease [52]. Lack of complete overlap from our repeated DNA-binding experiments, but with an underlying concordance of GO and motif grammar, indicates that we need to consider whether variation between different DNA-binding experiments or platforms indeed reflects noise or alternatively different biologically relevant GRN ‘states’. These findings shed light on the topical issue of the poor reproducibility of DNA-binding experiments, typically assessed through simple overlap metrics [53,54].

Our knowledge-based models identified a number of previously described NKX2-5 PPIs (GATA, HAND and TBX factor families) as being important features, supporting our hypothesis that these data could be used to predict novel NKX2-5 protein interactors. However, the majority of our predictions had not been previously described to interact with NKX2-5. We went on to test these predictions using the Y2H assay and validated 50% of the tested TFs as true NKX2-5 protein interactors: RXRα, PRRX2 and IKZF1/LYF-1. Of the PPIs that did not validate (TFAP4/AP-4, ZEB1 and MyoD), we found that this could be explained by motif redundancy. For example, the ZEB1/AREB6 motif is the reverse complement of that for FOXO4 (M00472) (figure 3*a*,*b*). FOXH1, a FOXO4 paralogue, has been previously described to interact with NKX2-5 [55]. MyoD could not be tested using the Y2H system, having a large amount of non-specific activity in the controls (data not shown). However, the MyoD and TFAP4 motifs clustered together and represent the canonical E-Box ‘CANNTG’ recognized by basic-helix–loop–helix (bHLH) proteins (figure 3*a*,*b*). It is possible that other bHLH TFs, such as HAND proteins, bind to the detected motif and interact with NKX2-5 through PPIs, as shown previously for HAND2 [56]. For confirmed novel PPIs, we also tested their paralogues, which we hypothesized could share the same motif. PRRX1a and PRRX1b, paralogues of PRRX2, as well as IKZF3 and IKZF5, paralogues of IKZF1, were confirmed as NKX2-5 interactors. However, we did not validate RXRγ, a paralogue of RXRα, suggesting that overall homology or the presence of PPI domains within paralogues is not necessarily a predictor of binding. This is consistent with previous findings that NKX2-5 could interact with NF1-B1 and NF1-B3 but not paralogous factors NF1-A or NF1-X [16]. The evolutionary significance of these PPIs in the context of NKX2-5 cardiac developmental biology and the conservation of their predicted genomic sites of interaction would benefit from further study.

Of the novel NKX2-5 PPIs predicted and validated, the homeodomain protein PRRX2 and retinoic acid receptor RXRα are expressed in the heart and play a role in heart development [42,57]. IKZF1 has not been associated with cardiac development and is better known for its role in haematopoietic differentiation, tumour suppression and chromatin regulation [58–60]. However, in the developing embryo, heart and haemoangiogenic progenitor territories have close physical relationships and share network regulators, which can act supportively as well as antagonistically to define territory boundaries [61–65]. Thus, it is conceivable that NKX2-5 and IKZF1 interact in the establishment and/or maintenance of these lineages, although further work is required to examine this. Both RXRα and IKAROS (LYF-1) are novel NKX2-5 PPIs that contain zinc-finger domains. NKX2-5 has been demonstrated to interact with other zinc-finger domain proteins, such as GATA4 [34], ZAC1/PLAGL1 [66] and CAL/FBLIM1 [67]. However, as observed in our Y2H assays, zinc-finger proteins ZBTB11/12 and KLF10 did not interact, so binding to zinc-finger domain proteins is not a generic feature of NKX2-5.

Perturbation of vitamin A (retinol) levels has long been known to affect mammalian embryo development, with the heart being the most sensitive organ [68,69]. Retinoic acid (RA), a derivative of vitamin A, is an essential signalling molecule that controls many aspects of embryo development by binding to RA receptors (RAR) and Retinoid X receptors (RXR). Changes in RA concentrations in retinal dehydrogenase (*Raldh2*)-deficient embryos leads to severe cardiac abnormalities [70], and removal of *Nkx2-5* in *Raldh2*^−/−^ mice rescues some of these defects [71], suggesting genetic cross-talk between the RA and NKX2-5 pathways. Mice lacking RXRα in the germline or conditionally in epicardium [72] display a spectrum of cardiac defects arising from lack of expansion of the myocardial compact zone and dysregulated trabecular morphogenesis [42]. Defects in mice lacking *Rxrα* and *Raldh2* overlap with those reported in *Nkx2-5* heterozygous and hypomorphic mice, raising the possibility that physical interaction between NKX2-5 and RXRα at early stages of heart development could be important for orchestrating normal morphogenesis. Disruption of *RXRα* was recently associated with the cardiac malformation tetralogy of Fallot [73], previously associated with mutations in *NKX2-5* [46,74]. We tested interactions between RXRα and five disease-causing NKX2-5 homeodomain mutants [46–48], observing a weaker interaction between RXRα and three of these (Q187H, R189G and R190H; figure 5). This work shows that NKX2-5 homeodomain mutations causative for CHD may critically intersect with RXRα pathways governing heart morphogenesis. Future studies assessing the role of these novel NKX2-5 protein interactions during normal development, evolution and in the context of disease models will further allude to their functional significance.

## 4. Conclusion

Using a knowledge-based machine-learning approach, we identified and validated a number of novel NKX2-5 protein interactors, RXRα, PRRX2 and IKZF1/LYF-1, and their paralogues PRRX1a, PRRX1b (two isoforms of PRRX1), and IKZF3 and IKZF5. Furthermore, we have established a potential CHD mechanism, whereby chamber and septal defects seen in patients carrying heterozygous NKX2-5 homeodomain mutations may in part be due to disrupted PPIs between NKX2-5 and RXRα. As far as we are aware, this is the first study to systematically validate predicted PPIs of TFs from DNA sequence alone.

Our study brings to light some key considerations. Comparing replicated experiments using a model-based approach supported our previous findings of conserved gene ontologies and indicated that motif grammar of NKX2-5 binding was conserved in repeated experiments. Thus, variation of binding sites identified between repeated experiments is not simply noise. In the light of NKX2-5 being a highly studied and critical TF for heart development, we identified and validated novel NKX2-5 PPIs from genome-wide DNA-binding data, demonstrating the utility of machine-learning approaches for systematic detection of TF binding partners. We propose that these interactions represent but a small proportion of the complex NKX2-5 PPI landscape that is difficult to probe using traditional methods. Identifying novel TF-TF PPIs has the potential to shed light on the complex gene regulatory processes underlying normal development and, as we observed for RXRα, provide new insights into disease processes.

## 5. Material and methods

Bioinformatics analyses were performed in R v. 3.1.2 (www.r-project.org) [75] using Bioconductor [76] packages unless stated otherwise.

### 5.1. Datasets

BED files corresponding to the mm9 coordinates of N-terminal NKX2-5 DamID peaks were downloaded from NCBI GEO Accession, GSE44902 [16]. We name repeated experiments, NKX2-5_1_ [GSE44902, GSM1093634] and NKX2-5_2_ [GSE44902, GSM1328466]. A random dataset was generated of the same set size and length distribution as NKX2-5 peaks using the permutation strategy implemented in bedtools [77] and sampling constrained to promoter regions represented on the microarray. Data were randomly partitioned for each NKX2-5 and random dataset into 75% for training and 25% for testing.

### 5.2. Motif detection and counting for generating feature matrices

DREME [23] was used for de novo motif discovery using only training sets. All motifs discovered according to default settings were reported. As DREME uses a one-way Fisher's exact test, we performed pairwise comparisons for NKX2-5_1_, NKX2-5_2_ and random peaks.

For generating motif feature matrices, we first add the PWMs of the de novo motifs discovered using DREME to a motif (PWM) library derived from Transfac and Jaspar public repositories, in addition to motifs from literature as previously described [16]; *n*(motifs) = 1132, bringing the total number of motifs used for analysis to 1202. All motifs are provided in electronic supplementary material, file S2.

CLOVER [27] was used to score PWM matches and each peak normalized to motif per kilobase to account for differences in motif numbers and length. A motif instance was recorded if it had at least the default minimum CLOVER score of 6. Formatting of data into feature matrices for input into R was performed using custom Perl scripts. Feature matrices, *M*_np_, used for classification were comprised of *n*_peaks_ by *p*_motifs/kb_.

### 5.3. Machine-learning algorithms and performance assessment

For generating lasso models, we used the ‘glmnet’ R library (v. 1.9) [78]. The lasso selects features by shrinking less relevant coefficients to zero through application of a *λ* penalty (via L-1 regularization; the shrinkage parameter). Ten-fold cross-validation was used to determine the value of *λ* and unless stated otherwise we used the *λ* within 1 s.e. of the maximum AUC. For SVM models, we used the ‘e1071’ R library (v. 1.6) [79] and the linear kernel function. The penalization parameter, *C*, was tuned using 10-fold cross-validation and a grid search space of 10^−5^ to 1 (identifying a C of 10^−4^ for models with and 10^−3^ without de novo motifs). For random forests, we used the ‘randomForest’ R library (v. 4.6) [80] with default parameters. For generating receiver operator characteristic (ROC) curves and calculating AUC, we used the ROCR package (v. 1.0) [81]. Sensitivity and specificity analysis considered the proportion of correctly classified true positive and true negative peaks as per equations (5.1) and (5.2):5.1
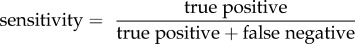
5.2
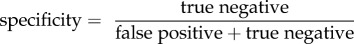


The positive predictive value (PPV) was calculated as follows:5.3
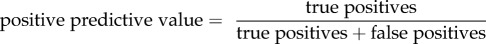


The false discovery rate (FDR) of predictions was calculated as follows:5.4
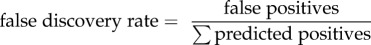


### 5.4. Yeast two-hybrid assay

Sequences coding for murine TFs MSX1, GMEB1, ZBTB11/12, KLF10 and NFYC were amplified from HL-1 cell cDNA. CMV AP-4 was a gift from Robert Tjian (Addgene plasmid # 12101) [82]; pLuc-CDS was a gift from Kumiko UiTei (Addgene plasmid # 42100) [83]; pBABE puro human RXRα was a gift from Ronald Kahn (Addgene plasmid # 11441); PRRX2-pSG5 was a gift from Corey Largman (Addgene plasmid #21009) [84]. A coding sequence was obtained from Origene for IKZF1 (MR227509), IKZF3 (MR227380), PRRX1a (RC213276), PRRX1b (RC210393) and RXRγ (MR225349). The vectors NpGBT9-AiolosF5-6 (M1-1 B9), NpGBT9-Eos-364-400 (M1-1 E9), pGBT9-Eos364-518 (M1-1 H6), pGBT9-Eos358-532 (M1-1 H8), NpGBT9-Pegasus (M1-1 I3), NpGBT9-Pegasus221-420 (M1-2 A1) and NpGBT9-PegasusF4-5 (M1-2 A2) were a gift from Merlin Crossley [85].

All sequences were cloned into pGADT7 AD or pGBKT7 DBD expression vector backbones (Clontech), which were modified to contain a Gateway cloning cassette (gift from Jacqueline Stoeckli). pGADT7-AD and pGBKT7-DBD fusions were co-transformed into chemically competent *S. cerevisiae* strain AH109 (Clontech). Double transformants were selected for growth on ‘low stringency’ -Leu/-Trp selection plates, before being selected for interaction on ‘high stringency’ -Ade/-His/-Leu/-Trp selection plates.

The monoclonal antibody 9E10 developed by Michael J. Bishop was obtained from the Developmental Studies Hybridoma Bank, created by the NICHD of the NIH and maintained at the Department of Biology, University of Iowa, Iowa City, IA 52242, USA.

### 5.5. Western blots

For protein extraction and western blotting, yeast colonies selected on ‘low stringency’ (-Leu/-Trp) plates were grown in 1.5 ml of liquid ‘low stringency’ medium at 30°C for 48 hours under agitation. Cultures were then transferred directly to 10 ml of fresh yeast extract protein peptone dextrose (YEPD) medium and further grown at 30°C for 4–6 hours under agitation until the OD_600_ reached 0.4–1.0. Protein extraction was then performed following the post-alkaline extraction method [86]. In accordance with this method, cultures were pelleted and resuspended in 100 µl of distilled water per 2.5 OD_600_. Then, 100 µl of 0.2 M NaOH was added per 2.5 OD_600_ and suspensions were incubated at room temperature for 5 minutes. After centrifugation, yeast cells were lysed in 50 µl of SDS sample buffer (0.06 M Tris-HCl, pH 6.8; 5% glycerol; 2% SDS; 4% β-mercaptoethanol; 0.0025% bromophenol blue) per 2.5 OD_600_ and boiled for 2 minutes. 20 µL of lysed samples were loaded on NuPage 10% bis-tris gels (Invitrogen).

To detect GAL4-activation (AD)-HA fusion proteins, a rabbit anti-HA antibody was obtained from Cell Signalling (C29F4). To detect the GAL4-DNA-binding domain (DBD)-c-Myc protein fusions, the monoclonal antibody 9E10 developed by Michael J. Bishop was obtained from the Developmental Studies Hybridoma Bank, created by the NICHD of the NIH and maintained at The University of Iowa, Department of Biology, Iowa City, IA 52242. After chemiluminescent detection, membranes were stained using a Ponceau S solution to visualize the total protein levels in each lane and control for equal loading. ImageJ (Rasband, W.S., US National Institutes of Health, Bethesda, Maryland, USA, http://imagej.nih.gov/ij, 1997–2016) was used to quantify protein expression detected by western blotting.

## Supplementary Material

Supplementary File 1

## Supplementary Material

Supplementary File 2

## Supplementary Material

Supplementary Table 1

## Supplementary Material

Supplementary Figures

## Data accessibility

Datasets are available at NCBI GEO: GSE44902 (GSM1093634) and GSE44902 (GSM1328466) and supporting data have been uploaded as part of the electronic supplementary material.
