# Supplementary material for: Prediction and validation of protein–protein interactors from genome-wide DNA-binding data using a knowledge-based machine-learning approach
Source: Open Biol. 2016 Sep 28;6(9):160183. doi: 10.1098/rsob.160183 (PMC5043580; doi:10.1098/rsob.160183)
Supplement: Supplementary Figures [file rsob160183supp4.docx]

# Prediction and Validation of Protein-Protein Interactors from Genome-wide DNA

# Binding Data using a Knowledge-based Machine Learning Approach

# Ashley J. Waardenberg, Bernou Homan, Stephanie Mohamed, Richard P. Harvey, Romaric Bouveret

# Supplementary Figures:

#
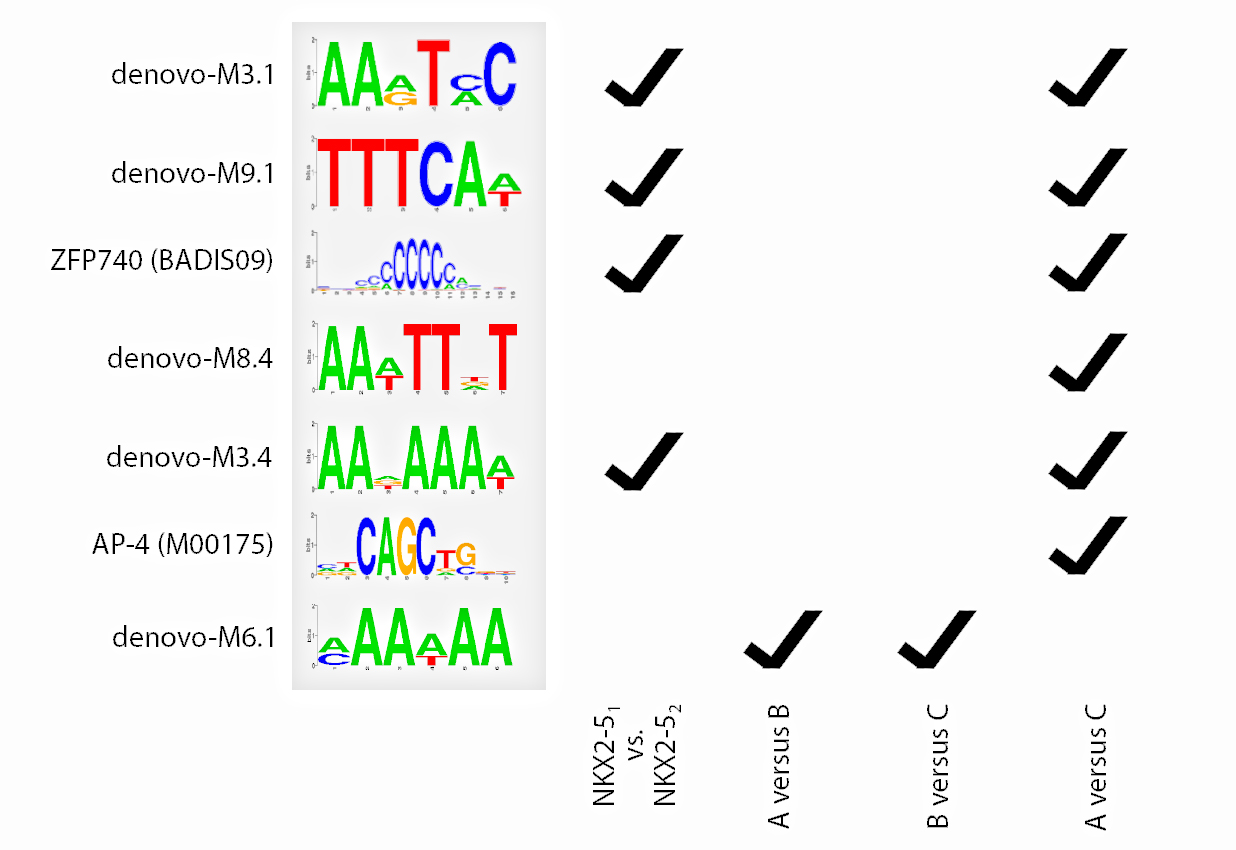


**Supplementary Figure 1.** Motifs identified in models directly comparing NKX2-5_1_ and NKX2-5_2_.


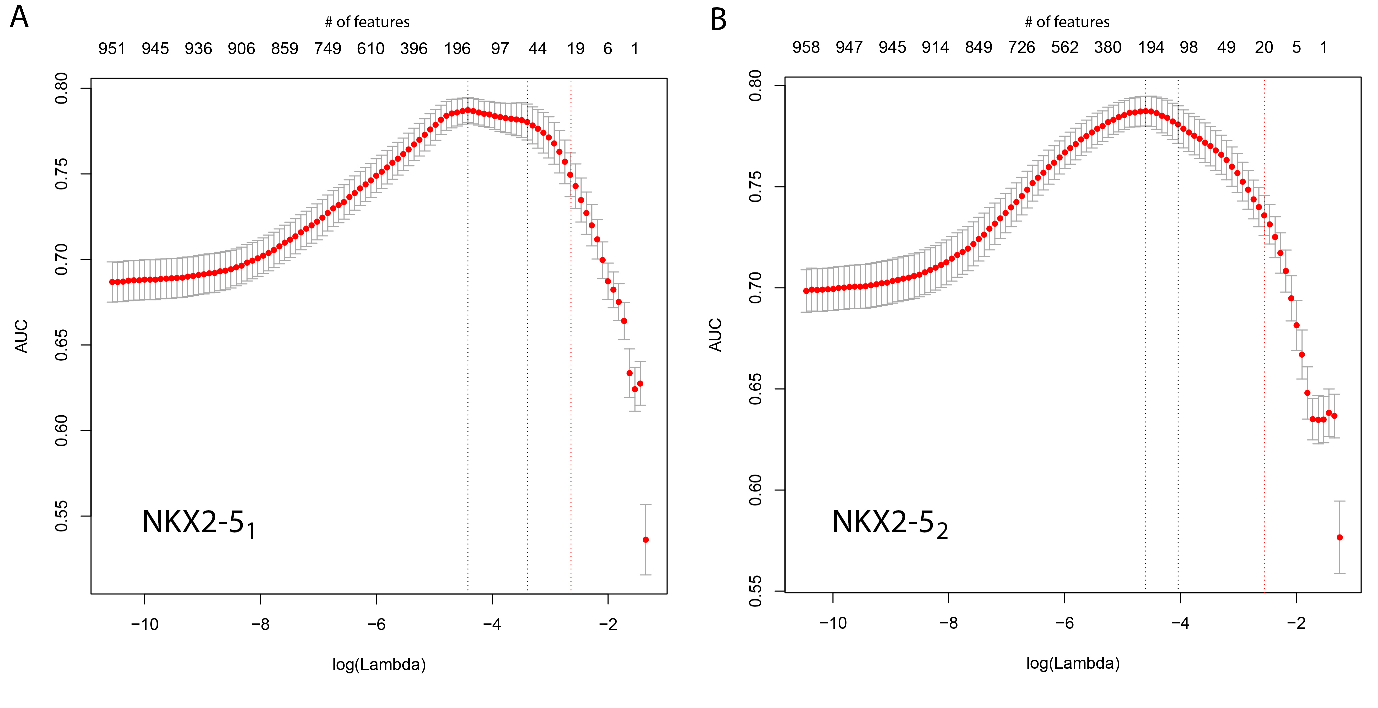


**Supplementary Figure 2.** Lamba curves for (A) NKX2-5_1_ and (B) NKX2-5_2_ relative to random. For each panel the most left hand side dotted black line indicates the lambda at maximum AUC, the next dotted black line to the right indicates the lambda at 1SE away from the maximum AUC and the red dotted line indicates the lambda selected using the knowledge-based approach. X-axis indicates Y-axis lambda value. Y-asis indicates AUC. Model size (number of features) is shown on the top of each graph. Error bars are resultant of 10 fold cross validation.

**
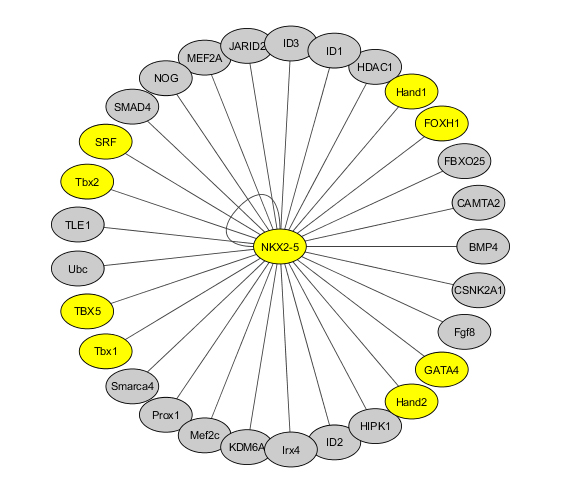
**

**Supplementary Figure 3.** Known NKX2-5 PPIs from public PPI databases (IntAct, HPRD, STRING, and BioGRID) corresponding to Supplementary Table 1. Yellow indicates protein families identified in our models.


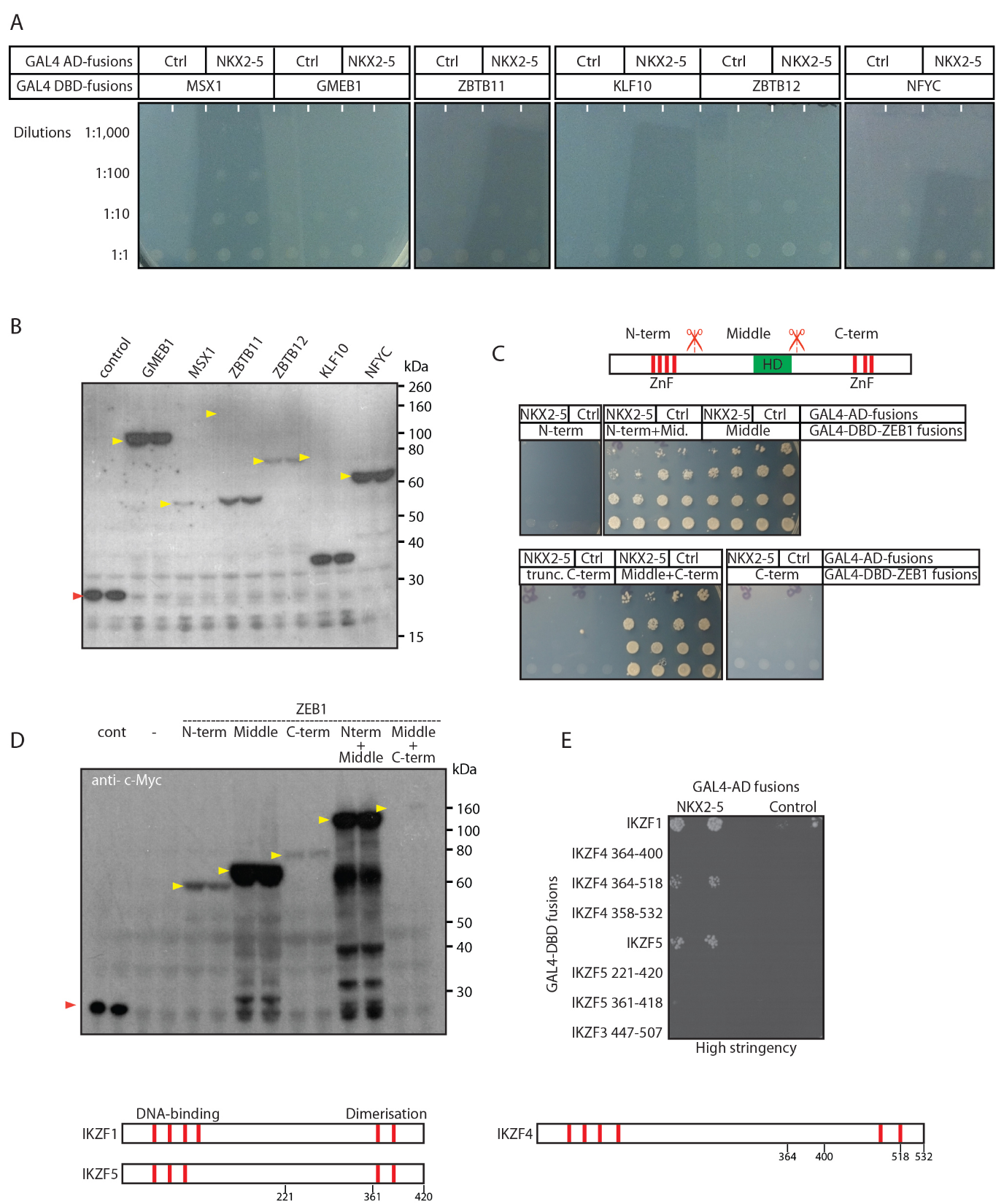


**Supplementary Figure 4.** Testing PPIs by yeast-2-hybrid. A. Representative pictures of yeast transformed with the GAL4-activation domain (AD) alone, Ctrl, or fused to NKX2-5 and the GAL4-DNA binding domain (DBD) alone or fused to negative-control protein interactors on plates containing selective medium. B. Detection of GAL4-DBD-Myc fusions by western blotting with anti-Myc antibodies. Coloured arrowheads indicate the expected molecular weight of control (red) or negative control interactors (yellow). C. Representative pictures of yeast transformed with the GAL4-AD alone (Ctrl) or fused to NKX2-5 and the GAL4-DBD alone or fused to fragments of the ZEB1 protein on plates containing selective medium. D. Detection of GAL4-DBD-Myc fusions by western blotting with anti-Myc antibodies. Coloured arrowheads indicate the expected molecular weights for control (red) or ZEB1 fragments (yellow). E. Representative pictures of yeast transformed with the GAL4-AD alone (control) or fused to NKX2-5 and the GAL4-DBD alone or fused to fragments of the IKAROS family members.
